# Mayer-Rokitansky-Kuster-Hauser syndrome associated with rectovestibular fistula

**DOI:** 10.4274/tjod.94809

**Published:** 2017-03-15

**Authors:** Charu Tiwari, Hemanshi Shah, Mukta Waghmare, Kiran Khedkar

**Affiliations:** 1 Topiwala National Medical College & Bai Yamunabai Laxman Nair Hospital, Mumbai, India

**Keywords:** Mayer-Rokitansky-Kuster-Hauser syndrome, atypical, rectovestibular fistula

## Abstract

A female neonate with two openings in the introitus and an absent anal opening at the anal site presents a diagnostic challenge. Mayer-Rokitansky-Kuster-Hauser (MRKH) syndrome associated with rectovestibular fistula, though rare, should be kept in mind as a differential diagnosis of this presentation. We present such a case in a one-year-old female child with MRKH syndrome and rectovestibular fistula.

## INTRODUCTION

Mayer-Rokitansky-Kuster-Hauser (MRKH) syndrome is a rare anomaly in females that affects 1 in 5000 live births^(1,2)^, in which there is dysgenesis of the Müllerian ducts leading to failure of development of the uterus and vagina. Ovarian function is preserved and the external genitalia are normal. The karyotype is 46, XX. There is normal development of secondary sexual characteristics at puberty. Primary amenorrhea at adolescence is the most common presenting symptom in patients with MRKH syndrome. However, when associated with anorectal malformation, this condition presents early at birth or in infancy and requires proper management^(3)^.

MRKH syndrome has been divided into two types (Schmid-Tannwald and Hauser, 1977); type A, or the typical form, is an isolated anomaly also known as the Rokitansky sequence^(4,5)^. The patient has symmetrical uterine remnants and normal fallopian tubes^(5)^. Type B, the atypical form, is characterized by asymmetric uterine buds or abnormally developed fallopian tubes (CAMP). This atypical form is associated with anomalies that involve other systems, especially the renal, cardiac, otologic, and skeletal systems^(1,4)^. Anorectal malformations are uncommonly reported to be associated with MRKH syndrome, and among them, rectovestibular fistula and cloacal malformations have been commonly described^(1,2,6)^.

We present a girl aged one year with atypical MRKH syndrome associated with rectovestibular fistula.

## CASE REPORT

A girl aged one year presented with an absent anal orifice since birth. She had been passing stools from an orifice within the introitus since birth. There was no constipation or abdominal distension. Per abdominal and systemic examinations were unremarkable.

**Figure 1:**
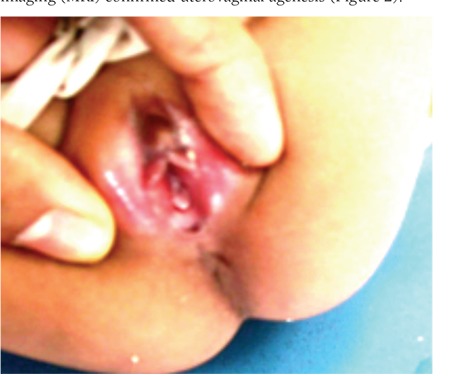
The perineum of the patient showing two orifices in the introitus

On perineal examination, there were two openings in the introitus. There was no anal opening at the normal site (Figure 1). The anterior opening was small and the child was passing urine through this opening, which suggested a urethral opening. The posterior opening in the vestibule discharged fecal matter, thereby suggesting a fistula. No vaginal opening could be appreciated.

Abdominal ultrasound revealed absent uterus and vagina. Both ovaries were normal and the right kidney was small. Barium enema showed a dilated rectum. Voiding cystourethrography was normal. A radio nucleotide renal study suggested a non-functioning right kidney and adequately functioning left kidney.

**Figure 2:**
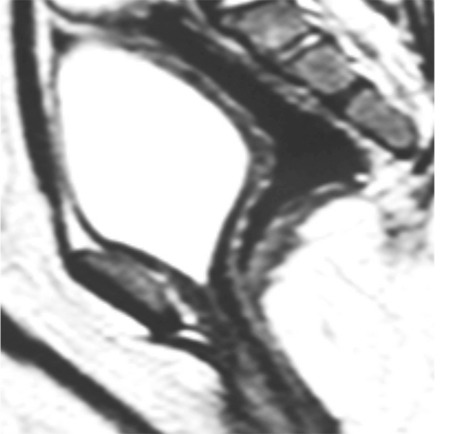
Magnetic resonance imaging pelvis showing uterovaginal agenesis and the dilated rectum

Cystogenitoscopy showed normal urethra and bladder, absent vagina, and the presence of rectovestibular fistula confirming the diagnosis of uterovaginal agenesis (MRKH syndrome type B). A right transverse stoma was performed. Magnetic resonance imaging (MRI) confirmed uterovaginal agenesis (Figure 2).

**Figure 3:**
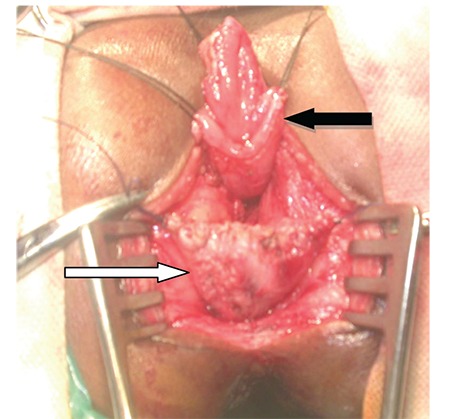
Intra-operative image of the patient showing the neovagina (retained distal end of rectum opening in the vestibule) (white arrow) and the pulled down bowel (black arrow)

Posterior sagittal anorectoplasty (PSARP) with a neovagina creation using the distal end of rectum with vestibular opening was planned. Approximately 3-4 cm of the distal ano-rectum (i.e. the rectovestibular fistula itself) was retained as a neo-vagina and the proximal rectum was brought down posteriorly within the sphincter complex (Figure 3).

At follow-up after 6 weeks, a neovagina of about 6-cm length along with minimal mucus discharge was present. The colostomy was closed after 8 weeks.

## DISCUSSION

The clinical appearance of two orifices in the introitus with an absent anal opening leads to the differential diagnosis of anorectal agenesis without fistula, a rectovaginal fistula (high or low) or a rectovestibular fistula with either a urogenital sinus or MRKH syndrome. The association of rectovestibular fistula with MRKH syndrome is rare with few reports in the literature^(1,2)^. Levitt et al.^(6)^, Gross^(7)^, Ein and Stephens^(8)^ reported 8, 2, and 2 such cases respectively^(1)^. Mahajan et al.^(9)^ described MRKH syndrome associated with H-type anovestibular fistula in 2009^(9)^. Ein and Stephens^(8)^ in 1971 first reported preservation of the rectum as a neovagina^(1)^. Gupta et al.^(1)^ recently reported this method of neovaginal reconstruction in a girl aged one year.

The etiology of MRKH syndrome is unknown; however, it is believed that there is interruption in the embryologic development during the sixth or seventh gestational week^(10,11)^. The spectrum of malformations associated with atypical MRKH syndrome suggests a developmental field defect involving organ systems that are closely related during embryogenesis^(4,12,13)^. MRKH syndrome has been attributed to an initial affection of the intermediate mesoderm, consequently leading to an alteration of the blastema of the cervicothoracic somites and pronephric ducts^(4,12)^. Mutations of the WNT4 and TCF2 genes have recently been found associated with MRKH syndrome^(4,14)^.

The importance of the clinical examination of the perineum in a female neonate cannot be over-emphasized. This diagnosis of utero-vaginal agenesis should be made at birth itself. The clinical presentation with two openings in the introitus with fecal matter deflating through the posterior opening requires investigations to confirm diagnosis before proceeding to the definitive management. Ultrasonography, a contrast study through the opening in the vestibule, MRI, and cystogenitoscopy through both openings in the introitus help in the definitive diagnosis of absent vagina and cervix^(4)^. This is essential for planning the definitive management.

A possible scenario that should not be forgotten is that failure of the neonate to pass meconium through the second opening within 24 hours of birth leads to a colostomy because of the assumption of anorectal agenesis without fistula^(1,6)^. Due to this presumed misdiagnosis, at the time of definitive repair, the rectum would not be found because of the incorrect assumption that the rectum was the vagina^(1,6)^. However, a distal colostogram performed before the definitive repair would surely help in suspecting this malformation^(1,6)^.

There are two surgical options for the definitive repair in patients with MRKH syndrome with rectovestibular fistula^(1,6)^. In the first method, the fistula is mobilized, traditionally by either PSARP or anterior sagittal anorectoplasty approach, and fixed within the sphincter muscle complex at the proposed anal site and a neoanus is created^(1,6)^. A vaginoplasty is performed at later date in these patients^(1,15)^. This type of repair is well suited for patients in whom MRKH syndrome was not diagnosed at infancy and presented at adolescence with symptoms of primary amenorrhea^(16)^. Wang et al.^(17)^ reported three patients who presented with MRKH syndrome and rectovestibular fistula with imperforate anus and symptoms of primary amenorrhea and loose stools. A single-stage anorectovaginoplasty was performed in these patients with laparoscopic assistance in one patient^(17)^.

The second option is to preserve the fistula at the vaginal site and leave an approximately 10-cm distal stump as a neovagina and to pull the proximal colon through the sphincter muscle complex as the neo-anorectum^(1,3,6)^. However, this procedure can only be performed when the correct diagnosis of MRKH syndrome with rectovestibular fistula is made pre-operatively^(1)^.

This second option is the preferred technique because it is relatively simple to perform; there is no chance of damaging any neural innervations, and both the neovagina and neoanus are created in the same operation^(1)^. Neovaginas have not been reported to show tendency for stricture formation; sphincter tone is good and patients are continent^(6)^.

Levitt et al.^(6)^ used the PSARP approach for this procedure. The abdominoperineal approach is required when the uterus is present to allow for the anastomosis of the rectal pouch (now the neovagina) to the uterus, thereby creating continuity of the reproductive system^(1)^.

The association of MRKH syndrome with rectovestibular fistula is rare and should be suspected as the differential diagnosis in a female neonate with two openings in the introitus. A correct pre-operative diagnosis helps to correct both malformations in the same operative procedure. Early diagnosis and simultaneous vaginal reconstruction and anorectoplasty in infancy offers added advantages; it prevents psychological trauma and avoids the need of delayed vaginoplasty through scarred perineum in these patients.
